# Relationships of 25-hydroxyvitamin D levels and non-alcoholic fatty liver disease in obese children: A possible strategy to promote early screening of NAFLD

**DOI:** 10.3389/fnut.2022.1025396

**Published:** 2022-11-02

**Authors:** Jeanette Irene Christiene Manoppo, Vivekenanda Pateda, Cindy Prayogo, Fima L. F. G. Langi, Fahrul Nurkolis, Apollinaire Tsopmo

**Affiliations:** ^1^Department of Pediatrics, Faculty of Medicine, Sam Ratulangi University, Manado, Indonesia; ^2^Department of Pediatrics, Prof. R. D. Kandou General Hospital, Manado, Indonesia; ^3^Faculty of Medicine, Sam Ratulangi University, Manado, Indonesia; ^4^Department of Biological Sciences, Faculty of Sciences and Technology, State Islamic University of Sunan Kalijaga (UIN Sunan Kalijaga Yogyakarta), Yogyakarta, Indonesia; ^5^Department of Chemistry, Carleton University, Ottawa, ON, Canada

**Keywords:** child, NAFLD, liver, obese, 25-hydroxyvitamin D, vitamin D

## Introduction

Intake of sufficient concentrations of vitamins is directly related to good health status. The diverse physiological roles of vitamins are generally grouped into water-soluble (or B-vitamins) and fat-soluble vitamins. All B vitamins are essential for cell functions such as glycolysis, gluconeogenesis, fatty acid metabolisms, and amino acids (1). Most vitamins also act as signaling molecules through phosphatases, kinases, or transcription factors (2). Some of the vitamins (e.g., vitamins C, E, and A) have received wide attention in part because of their antioxidant activities via radical scavenging, regulation, and antioxidant enzymes or antioxidant reactive elements mechanisms (3–5). Many effects of vitamins on health are still widely studied, such as the role of vitamins in acute respiratory distress syndrome and their microbiota regulation properties (6). One of the vitamins with newly discovered functions (e.g., a link with fatty liver) is vitamin D, which is one of the four fat-soluble vitamins. It is well characterized by its role in controlling the metabolism of calcium and phosphate. The consequence is healthy mineralization of the bones and regulation of the immune system. The active form of vitamin D (1,25-dihydroxy vitamin D) has been demonstrated in experimental experiments to exhibit immunologic effects on several constituents of the immune system and the integrity of the endothelial membrane. Low blood 25-hydroxyvitamin D levels were associated with an elevated risk of developing some immune-related conditions and diseases, including diabetes, multiple sclerosis, rheumatoid arthritis, respiratory infection, and nonalcoholic fatty liver disease (NAFLD) (7).

The insufficiency of vitamin D frequently coexists with NAFLD, and they share many cardiometabolic risk factors (8). This fact is supported by Liu et al. (9), who showed that a lower amount of vitamin D is an independent risk factor for NAFLD. In addition, the most prevalent long-term liver disease in children is pediatric NAFLD, whose incidence is growing along with increasing rates of obesity and overweight (10). These conditions call for nutritional intervention, as evidenced by a significant improvement in clinical markers of NAFLD in people who received vitamin D supplementation (11, 12). Therefore, the main objective of this article is to analyze the most recent research on the connection between NAFLD and vitamin D in obese children to emphasize the need for early screening for NAFLD in this population.

## Vitamin D deficiency and diseases

Vitamin D is a fat-soluble micronutrient that has two equal forms, namely vitamin D2 and vitamin D3, which are biologically inert. Vitamin D2 is obtained from the diet as ergosterol, primarily from mushrooms and fungi, and is then transformed to ergocalciferol by ultraviolet light, while vitamin D3 (cholecalciferol) is endogenously produced in the skin through the effect of UV-B on 7-dehydrocholesterol (13). Once absorbed from the intestine, vitamins D2 and D3 are metabolized in the liver and then form 25-hydroxyvitamin D [25(OH)D], consisting of 25(OH)D2 and 25(OH)D3. Vitamin 25(OH)D (also called calcidiol) is further converted so that a 1,25-dihydroxy vitamin D 1,25(OH)2D is formed. Vitamin D has both skeletal (calcemic) and extra-skeletal (non-calcemic) functions. The skeletal function is the function of vitamin D in terms of maintaining bone mineral homeostasis and bone growth, while the non-calcemic function is its function in modulating the innate and adaptive immune systems (14).

Vitamin D is often associated with several diseases, from infectious diseases to malignancies. Higher serum vitamin D levels are suggested to regulate calcium homeostasis and reduce the risk of obesity, insulin resistance, metabolic syndrome, and malignancy (15). Vitamin D deficiency may be caused by decreased dietary intake or impaired absorption, decreased sun exposure, decreased endogenous synthesis, and end-organ vitamin D resistance due to hereditary issues (16). It has also been demonstrated that personal traits and the environment have an impact on how vitamin D is synthesized in the skin (17). While obesity has been defined as a risk factor for vitamin D deficiency due to metabolic impairment and adiposity status (18), obese children also spend less time playing outside and more time indoors (19), reducing sun exposure related to vitamin D synthesis. Surprisingly, 10.8% of children in South China have vitamin D deficiency, while vitamin D insufficiency reaches 39% (20). The prevalence of hypovitaminosis D in America is 60.4%, with an insufficiency of 44.6% and a deficiency of 15.8% (21). In Indonesia, vitamin D insufficiency (25–49 nmol/L) reaches 45.1%, while the prevalence of insufficiency (50–74 nmol/L) and sufficient (≥75 nmol/L) reach 49.3% and 5.6% (22). In Jakarta, vitamin D insufficiency in elementary school children was 75.9% and vitamin D deficiency was 15%. Vitamin D insufficiency in Manado, North Sulawesi, is 34%, while vitamin D deficiency is 64% in adolescents aged 10–18 years (23).

## Non-alcoholic fatty liver disease in obese children

Non-alcoholic fatty liver disease is a long-term liver disease that occurs due to the accumulation of excess fat in the liver organs. Non-alcoholic fatty liver (NAFL), a more benign disease, and non-alcoholic steatohepatitis (NASH), a more severe illness, are both subsets of NAFLD (24). NAFLD in the pediatric population is defined as long-standing liver steatosis in children (≤ 18 years) thatis not caused by metabolic or genetic disorders, malnutrition, infections, ethanol consumption, or the use of steatogenic drugs (25, 26). Steatosis, as a sign of NAFLD, is formed due to the rate of taking fatty acids from the liver from plasma and de novo lipogenesis, which is much higher than the rate of oxidation of fatty acids. Excessive levels of intrahepatic triglycerides mark the presence of an imbalance between metabolic interactions. Steatosis is related to a combination of metabolic disorders of glucose, fatty acids, and lipoproteins. On the other hand, clinical conditions such as obesity, insulin resistance, and type 2 diabetes have all been linked to NAFLD (27). The unusual metabolism of fatty acids combined with elevated adipose tissue and liver-systemic inflammation is the key to the formation of risk factors for the occurrence of NAFLD (28). However, significant pathophysiological interactions between circulating lipids, adiponectin, muscle and liver tissues, pancreas, and gastrointestinal hormones in connection to nutrition, exercise, and inflammation also need to be considered when discussing the pathophysiology of NAFLD (29).

The prevalence of NAFLD in children reaches 7.6% of the general population and 34% in pediatric patients with obesity (30). The prevalence of children diagnosed with NAFLD has increased annually in the last 10 years (31, 32). The incidence of NAFLD incidence among children increased by 62%, that is, from 0.036% in 2009 to 0.0582% in 2018 (31). Obesity itself is closely related and is the major precursor of the appearance of NAFLD. The prevalence of NAFLD prevalence also has a positive relationship with body mass index (BMI). Analysis of liver histology showed that the picture of steatosis in obese patients reached 65−85%. Several other studies that have been conducted have found that a decrease in 25 (OH)D levels in the body increases the occurrence of NAFLD (33–36). Those studies found a negative association between vitamin D status with NAFLD, fibrosis, and NASH in adolescents and children. Furthermore, vitamin D insufficiency was also found to be more common in obese patients than in those of normal weight. Many findings on the prevalence of NAFLD are summarized in Table 1.

**Table 1:** Table of the proportion of cases of vitamin D insufficiency or deficiency in obese children with NAFLD and obese children without NAFLD.

**Studies**	**Total sample**		**Number of insufficiency/deficiency cases** **vitamin D**	
	**NAFLD**	**Non-NAFLD**	**NAFLD**	**Non-NAFLD**
Black et al. (37)	156	838	Insufficient: 79 (50.6%) Deficient: 27 (17.3%)	Insufficient: 316 (37.7%) Deficient: 93 (11%)
Malespin et al. (38)	12	134	Deficient: 10 (83.3%)	Deficient: 66 (49.2%)
Sezer et al. (39)	58	53	Insufficient: 17 (29.3%) Deficient: 39 (67.2%)	Insufficient: 13 (24.5%) Deficient: 40 (75.4%)
Cho et al. (40)	215	3,663	Deficient: 183 (85.1%)	Deficient: 2,835 (77.4%)

## Discussion

Lower serum levels of 25(OH)D in obese children are caused by the uptake of 25(OH)D in the body's adipose tissue, causing its bioavailability to decrease. Vitamin D plays an important role in inhibiting the adipogenesis process so that a decrease in its levels in the blood will trigger adipogenesis so that the synthesis process of adipocytes continues. Research shows that NAFLD is more prevalent in obese children. Elizabeth et al. (41) found that the incidence of NAFLD is more common in children with obesity. Anderson et al. (30) showed that the prevalence of NAFLD in healthy children was 7.6% (95%CI; 5.5−10.3%), while in obese children it was 34% (95%CI; 27.8−41.2%) (30). Obesity is a significant factor in the development of hepatosteatosis. Several theories were developed to understand the mechanism of the course of obesity that causes hepatosteatosis. One of the theories stated that an injury to hepatocytes may be caused by oxidative stress and insulin resistance (40). Kitade et al. (27) found that a high accumulation of lipids in the liver activates macrophages and Kupfer cells, causing an increase in insulin resistance, as well as liver inflammation and fibrogenesis.

**Figure 1:**
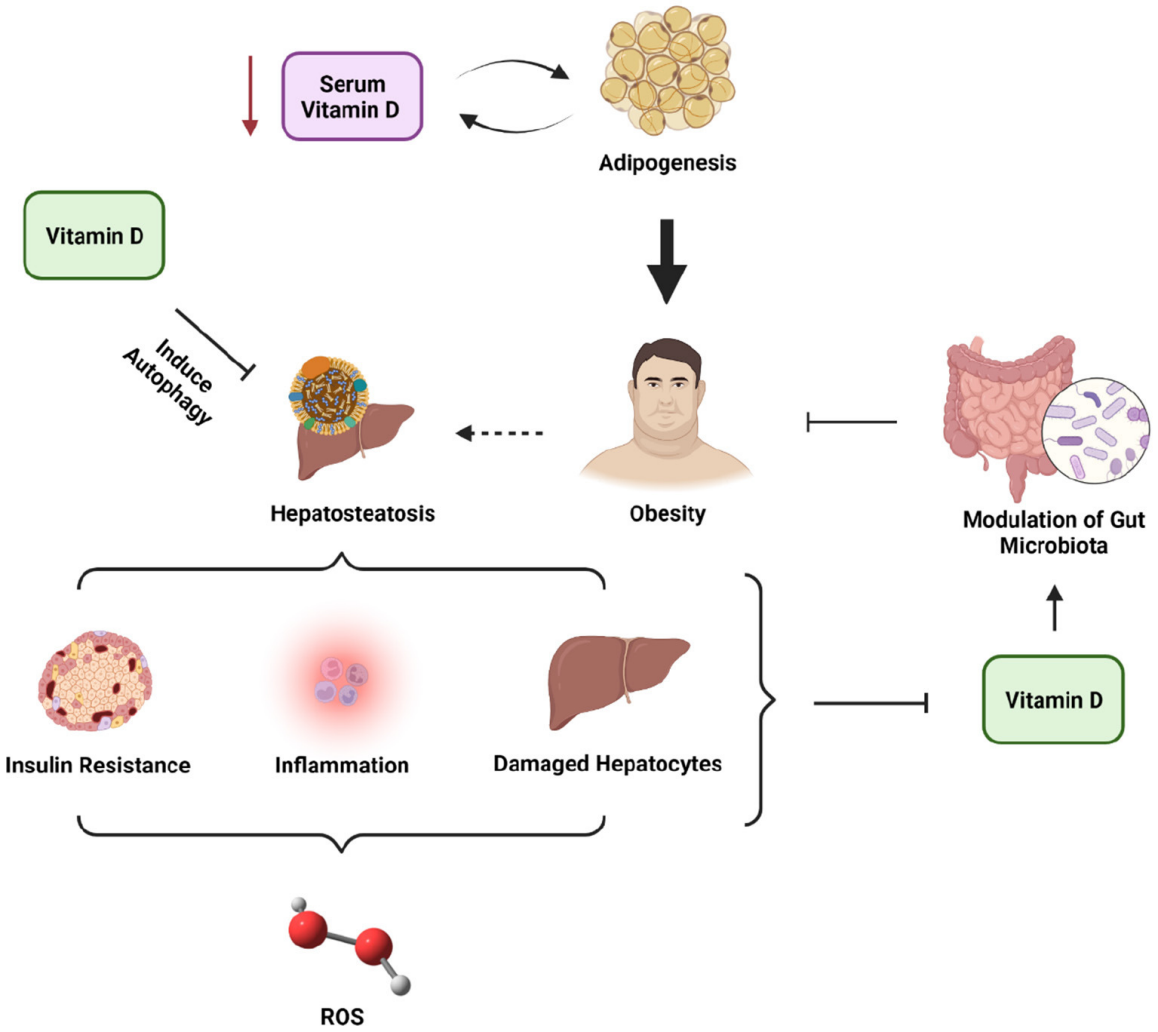
The roles of vitamin D in NAFLD and obesity.

Such a broad role of vitamin D has shown that, in addition to maintaining bone mineral homeostasis, vitamin D also plays an important role in cell proliferation, namely as an anti-inflammatory agent and immunomodulatory. However, the function of vitamin D is not restricted to those functions alone. Vitamin D can also protect insulin sensitivity and secretion. Insufficient vitamin D levels were evidenced to contribute to the development of insulin resistance and NAFLD and worsen NAFLD conditions through activation of TLR (toll-like receptor) due to endotoxin exposure, thus causing liver inflammation and oxidative stress (35, 42, 43) (Figure 1). Vitamin D has been shown to protect against liver steatosis caused by a high-fat diet (HFD) or free fatty acid (FFA) by activating autophagy in mice cells (44). Additionally, vitamin D was also known to modulate the gut microbiota, which may be beneficial in the pathophysiology of NAFLD (45, 46).

Another mechanism that also causes inflammation in the liver is due to a decrease in 25 (OH)D levels, namely by disrupting tight junctions and damaging the intestinal barrier so that there is a translocation of bacteria and their endotoxins to the portal circulation, which increases inflammation in the liver and triggers hepatocyte apoptosis (47) (Figure 1). Some exposure to the above mechanisms suggests that a reduction in 25(OH)D levels along with obesity increases the risk of NAFLD in children. Due to the “pleiotropic” effects of vitamin D, which include roles in immunomodulation and control of inflammation, vitamin D insufficiency has recently been associated with the etiology and complexity of NAFLD (48). The roles of vitamin D in ameliorating NAFLD and obesity conditions are presented in Figure 1. Interestingly, many studies could not establish a direct relationship between vitamin D levels and the incidence of NAFLD in children and adolescents (37–40), which may be explained due to the observational study designs in each study, especially cohort, cross-sectional, and case-control. Therefore, more solid evidence is needed to determine the direct contributors or mechanisms of NAFLD in children and adolescents.

## Future implications

The level of 25(OH)D in obese children with NAFLD is lower than in healthy children and obese children who do not suffer from NAFLD. For clinical purposes, we suggest that it is necessary to detect 25 (OH)D levels in obese children so that early treatment can be performed to prevent NAFLD. More cohort research and clinical trials are needed on vitamin D supplementation in obese children with NAFLD who experience vitamin D insufficiency or deficiency to improve NAFLD while also establishing the direct cause of NAFLD in children and adolescents.

## Author contributions

JM, CP, VP, FL, and FN contributed to the conceptualization with the design of the opinion study and drafted the manuscript. AT and FN edited-revised and approved the final version of the submitted manuscript. All authors and contributors contributed to the opinion article and approved the submitted version.

## Conflict of interest

The authors declare that the research was conducted in the absence of any commercial or financial relationships that could be construed as a potential conflict of interest.

## Publisher's note

All claims expressed in this article are solely those of the authors and do not necessarily represent those of their affiliated organizations, or those of the publisher, the editors and the reviewers. Any product that may be evaluated in this article, or claim that may be made by its manufacturer, is not guaranteed or endorsed by the publisher.
